# Three-Dimensional Magnetic Rehabilitation, Robot-Enhanced Hand-Motor Recovery after Subacute Stroke: A Randomized Controlled Trial

**DOI:** 10.3390/brainsci13121685

**Published:** 2023-12-07

**Authors:** Sung-Hoon Kim, Dong-Min Ji, In-Su Hwang, Jinwhan Ryu, Sol Jin, Soo-A Kim, Min-Su Kim

**Affiliations:** 1 Department of Electronics & Information Engineering, Korea University, Sejong 30019, Republic of Korea; kshoon04@korea.ac.kr; 2 Department of Electronics Convergence Engineering, Wonkwang University, Iksan 54538, Republic of Korea; anggole94@gmail.com; 3 Department of Rehabilitation Medicine, Soonchunhyang University Cheonan Hospital, Cheonan 31151, Republic of Korea; 135829@schmc.ac.kr (I.-S.H.); 129751@schmc.ac.kr (J.R.); 138411@schmc.ac.kr (S.J.); sooapmr@schmc.ac.kr (S.-A.K.); 4 Department of Regenerative Medicine, College of Medicine, Soonchunhyang University, Cheonan 31151, Republic of Korea

**Keywords:** hand, finger, magnets, rehabilitation, robotics, stroke, upper extremity

## Abstract

We developed an end-effector-type rehabilitation robot that can uses electro- and permanent magnets to generate a three-way magnetic field to assist hand movements and perform rehabilitation therapy. This study aimed to investigate the therapeutic effect of a rehabilitation program using a three-dimensional (3D) magnetic force-based hand rehabilitation robot on the motor function recovery of the paralyzed hands of patients with stroke. This was a double-blind randomized controlled trial in which 36 patients with subacute stroke were assigned to intervention and control groups of 18 patients each. The intervention group received 30 min of rehabilitation therapy per day for a month using a 3D magnetic force-driven hand rehabilitation robot, whereas the control group received 30 min of conventional occupational therapy to restore upper-limb function. The patients underwent three behavioral assessments at three time points: before starting treatment (T0), after 1 month of treatment (T1), and at the follow-up 1-month after treatment completion (T2). The primary outcome measure was the Wolf Motor Function Test (WMFT), and secondary outcome measures included the Fugl–Meyer Assessment of the Upper Limb (FMA_U), Modified Barthel Index (MBI), and European Quality of Life Five Dimensions (EQ-5D) questionnaire. No participant safety issues were reported during the intervention. Analysis using repeated measures analysis of variance showed significant interaction effects between time and group for both the WMFT score (*p* = 0.012) and time (*p* = 0.010). In post hoc analysis, the WMFT scores and time improved significantly more in the patients who received robotic rehabilitation at T1 than in the controls (*p* = 0.018 and *p* = 0.012). At T2, we also consistently found improvements in both the WMFT scores and times for the intervention group that were superior to those in the control group (*p* = 0.024 and *p* = 0.018, respectively). Similar results were observed for FMA_U, MBI, and EQ-5D. Rehabilitation using the 3D hand-rehabilitation robot effectively restored hand function in the patients with subacute stroke, contributing to improvement in daily independence and quality of life.

## 1. Introduction

The human hand is one of the most fascinating and sophisticated biological motor systems, and its complex biomechanics and neural architecture enable it to grasp objects of various shapes and sizes through the coordinated motions of multiple fingers that can engage in creative and practical activities, such as writing, drawing, and playing musical instruments [1]. Hand function also has huge implications for performing tasks in a person’s occupation. Greater difficulties in hand function correspond to increased impairment in the use of assistive technology enabling participation in academic and social activities [2]. Upper-extremity motor function impairment reportedly occurs in ≤80% of patients with stroke [3], and the extent of a patient’s upper-extremity dysfunction is determined by the degree of functional hand impairment [4]. Several rehabilitation techniques have been developed to restore impaired hand function after stroke, including constraint-induced movement therapy [5], repetitive transcranial magnetic stimulation [6], and traditional occupational therapy. Although these therapies have partially contributed to the recovery of hand function after stroke, the complexity and versatility of the human hand pose a major challenge in stroke rehabilitation [7].

In light of these challenges, clinicians and researchers have begun to actively apply robotic therapeutic techniques to patients undergoing stroke rehabilitation. Robots used to restore motor function in the upper limb are broadly categorized into end-effector-type robots and exoskeletal-type robots [8]. The end-effector-type hand-rehabilitation robot is connected to the distal part of the patient’s upper limb and can apply free-exercise programs according to the patient’s hand-function level [8]. Exoskeletal-type hand-rehabilitation robots have the joint axes of the robot aligned with the joint axes of the patient’s hand, and can train specific muscles by controlling joint movements with calculated torques [9]. Robotic-assisted hand rehabilitation is often used to improve motor function in stroke-related paralyzed hands and has shown significant therapeutic benefits compared with conventional treatment [10,11]. Wearable robots have gained attention as they can embody motor functions tailored to various hand movements by collecting motion data or physiological signal data on the user’s hand movements through device-mounted sensors [12]. These robots also reportedly have a positive effect on hand motor function recovery in patients with stroke [13]. Virtual-reality programs are additionally applied to improve patient compliance with the robot [14], and hand-rehabilitation robots are being developed with artificial intelligence technology to provide a variety of patient-specific protocols [15].

We have noted that magnetic forces can be efficiently used to assist the strength of hands paralyzed by stroke and to perform exercise therapy. Magnetic forces are invisible and can give patients the sensation that their fingers are actually moving, which can reduce resistance to treatment [16]. Moreover, the advantage of magnetic forces is that they can implement a variety of finger movements in different directions based on the magnetic force direction, regardless of the position of the hand [7]. We previously developed a three-dimensional (3D) hand-rehabilitation robot that can perform finger-rehabilitation training with constant force and orientation regardless of hand position and confirmed the short-term therapeutic effect in an earlier study [17]. However, we were still uncertain if the 3D hand-rehabilitation robot could contribute to the long-term recovery of hand function in patients with stroke. Therefore, this study aimed to investigate the long-term effects of a 3D hand-rehabilitation robot on the recovery of hand function in patients with stroke-related hand paralysis.

## 2. Materials and Methods

### 2.1. Magnetic Force-Driven Hand-Rehabilitation Robot

**Figure 1:**
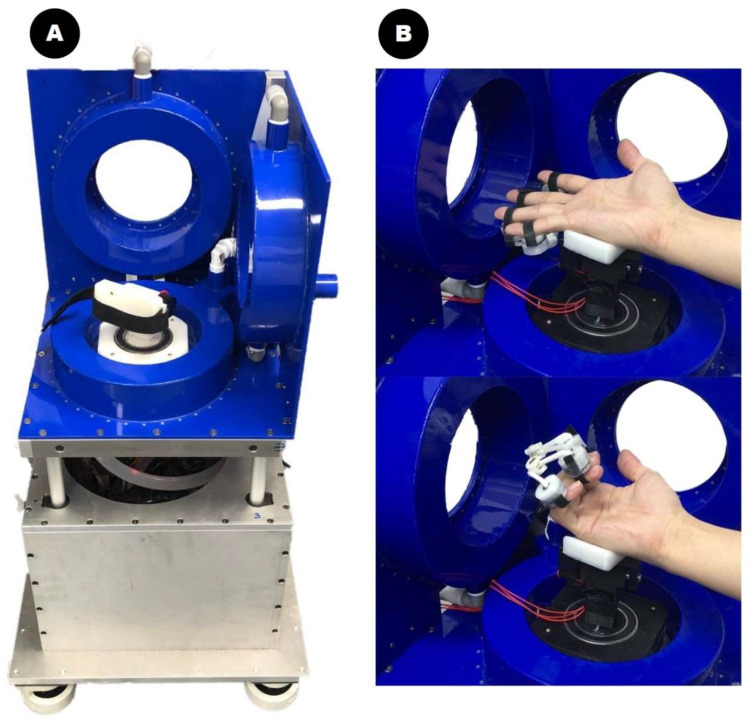
The three-dimensional magnetic force-driven finger-rehabilitation robot is shown. (**A**) The developed magnetic array device. (**B**) The extension and flexion movements of the hand aided by magnetic forces in the device. The magnetic array placed on the patient’s finger generates attraction and repulsive forces driven by the magnetic field of the three-dimensional coil system. These magnetic forces are used to move the paralyzed fingers of patients with stroke.

A developed electromagnetic rehabilitation system with multilink magnetic devices on the fingers can create and induce flexion and extension movements of the fingers because the applied alternating current (AC) magnetic field generates magnetic forces (attraction and repulsion) [16]. These forces create a bending or extending motion of the fingers. The magnetic force required to move the finger the desired amount is controlled by the amount of current flowing through the coils [18]. The 3D hand-rehabilitation systems with magnetic multilink devices have the advantage of being able to detect finger positions in real time, enabling active flexing and extending regardless of the hand position (Figure 1).

Because patients with stroke cannot remain immobilized for long periods of time, their finger positions are constantly changing. Therefore, the change in angle is fed back to the coil’s current controller, and the direction of the magnetic field is automatically changed by the control algorithm to match the hand position. The robot can effectively perform finger-rehabilitation exercises by applying a constant external force to the patients fingers at all times, regardless of the patient’s hand position. More details about the magnetic force-based hand-rehabilitation robot’s mechanism are presented in a previous paper [17].

### 2.2. Study Design

The study included patients with ≥grade 2 finger motor grade by manual muscle test on the paralyzed side after stroke. The patients’ ages ranged from ≤20–80 years. Stroke onset had occurred ≤3 months before study inclusion for all patients. The patients with spasticity or severe muscle shortening of a modified Tardieu Scale grade ≥3, patients with severe cognitive impairment who were unable to understand the physiotherapist’s instructions, maintain a sitting position, and receive appropriate rehabilitation due to serious medical conditions, such as pneumonia, were excluded from the study.

This was a parallel-group, single-blind, randomized controlled trial (Unique identifier: KCT0007970) with participants randomly assigned in a 1:1 ratio between the treatment and placebo groups. A block randomization process to ensure equal numbers in each treatment group was used by a statistician to achieve randomization before starting the trial. The participants were randomly assigned to the intervention and control groups.

The intervention was designed so that the control and experimental groups received the same amount of rehabilitation time. Patients of intervention and control groups equally received occupational therapy to restore upper limb function for 1 h a day. Specifically, the patients in the control group received conventional occupational therapy, including the upper-extremity range of motion exercises, finger stretching, sensory stimulation, and strengthening exercises for one hour once a day. The patients in the intervention group received conventional occupational therapy for 30 min, followed by magnetic force-driven robotic hand rehabilitation therapy for the remaining 30 min a day.

Physical therapy programs such as neurodevelopmental therapy, muscle strengthening exercises, and gait training, which are generally administered to stroke patients, were performed equally for both groups for an hour per day.

### 2.3. Magnetic Force Robot Finger-Rehabilitation Protocol

The intervention group received rehabilitation using a magnetic finger-rehabilitation device. The rehabilitation exercises with the device included (1) flexion/extension of fingers, (2) a sequential finger–thumb opposition exercise, and (3) twisting of metacarpophalangeal joint exercises. To stimulate proprioception in the hand and prevent shortening of the finger muscles, magnetic forces were used to perform finger flexion/extension. Finger–thumb counter movements using the thumb and the other four fingers were performed to aid in the functional movement of the pinch grip. Torsion of the metacarpophalangeal joint was applied to lengthen the distal hand joint.

All exercises were designed as active-assisted exercises to allow the participants to perform as many movements as possible with the help of magnetic force. If the participant needed more assistance due to muscle shortening, we controlled the magnetic force to perform the exercise. Each exercise using a magnetic finger-rehabilitation device was performed for 10 min, and one treatment session lasted approximately 30 min.

### 2.4. Behavioral Outcome Measures

The Wolf Motor Function Test (WMFT) was evaluated as the primary outcome to compare treatment effects between the intervention and control groups. The evaluator assessed the patient’s hand function without knowing to which group the subject was assigned. The WMFT consisted of 15 functional tests and two strength tests involving complex movements from proximal to distal interphalangeal joints that comprehensively assess upper-extremity motor function [19]. Each of the 15 assessment items measured the time required for a participant to fully perform a given task, with a maximum allowable time of 120 s. WMFT scores reflect the level of hand movement while performing various tasks [20], and scores range from 0 to 75, with higher scores indicating better hand motor function.

As secondary outcome measures, we used the (1) Fugl–Meyer Assessment of upper-extremity (FMA_U), (2) modified Barthel Index (MBI), and (3) European Quality of Life Five Dimensions (EQ-5D). For both the primary and secondary outcomes, the patients underwent three behavioral assessments: before the intervention (T0), after 1 month of treatment (T1), and 1 month after the treatment ended (T2). The physician who performed the behavioral assessment did not know to which treatment group a subject had been assigned.

Demographic information, including age, sex, stroke type, dominant hand, affected side, stroke onset to treatment period, rigidity degree, National Institutes of Health Stroke Scale (NIHSS) score at the time of emergency room admission for stroke, Montreal Cognitive Assessment (MoCA) score, and underlying medical conditions, such as diabetes and hypertension, were collected before treatment initiation.

### 2.5. Statistics

Sample size calculations were performed for this study based on the primary outcome, the WMFT score. To satisfy the alpha level of 0.05 with a power of 0.80, at least 14 subjects were needed in each of the two groups. Considering the dropout rate of 20%, a total of 18 subjects were required for each group.

Descriptive statistics were used for the participants’ characteristics and exercise test results. The Shapiro–Wilk test was performed for all quantitative variables to determine if the distribution was normally distributed. For the demographic data of the two groups, independent *t*-tests were performed for continuous variables and χ^2^ tests were performed for categorical variables. To compare changes in outcome measures, we used repeated-measures analysis of variance (rm-ANOVA), with time as the within-patient factor and group as the between-patient factor for normally distributed parametric data. The Bonferroni test method was used to perform post hoc tests, and values of *p* < 0.05 were accepted as indicating statistical significance. SPSS Statistics v.29.0 (IBM SPSS Statistics for Windows, IBM Corp., Armonk, NY, USA) was used to perform all statistical analyses.

## 3. Results

### 3.1. Safety Issue

Rehabilitation physicians, occupational therapists, and robotic-development engineers monitored possible side effects while the patients underwent rehabilitation therapy using the magnetic force-driven rehabilitation device. The patient’s sensory or proprioceptive deficits were also examined before the intervention began. The rehabilitation physician also checked to see if the patient had any soft-tissue injuries or musculoskeletal pain in the upper extremities before and after each intervention session. No participants raised any safety concerns, including injuries, during the intervention.

### 3.2. Demographic Characteristics

**Figure 2:**
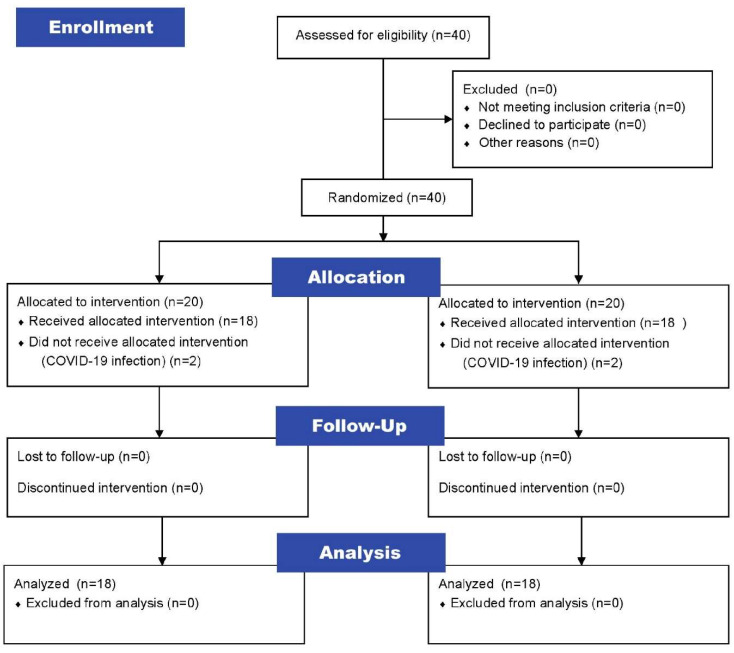
Participant-selection flow.

A total of 40 participants were recruited, and the intervention and control groups were each randomly assigned 20 patients. Four patients were excluded during the study period because they were all infected with COVID-19 and quarantined, so they were unable to receive the intervention. Consequently, there were 18 patients in the intervention group and 18 patients in the control group as the final participants in the study (Figure 2).

The mean age of the participants was 60.8 ± 10.4 years, and the mean time from stroke onset to treatment was 29.5 ± 8.4 days. There were no significant differences in the demographic and clinical characteristics between the two study groups (Table 1). In addition, the NIHSS and MoCA scores, spasticity severity, and co-morbidities were not significantly different between the intervention and control groups.

### 3.3. Primary Outcome Measures

**Figure 3:**
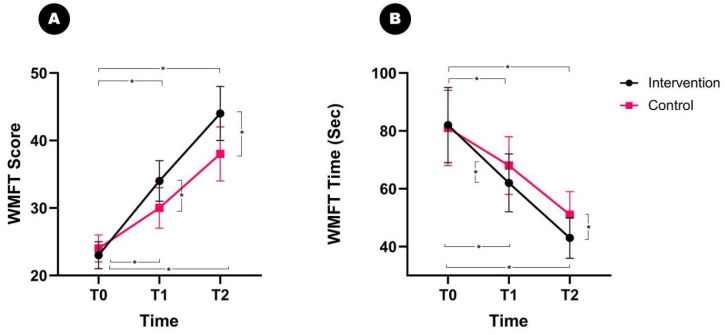
Changes in WMFT after treatment with a magnetic force hand-rehabilitation robot. The WMFT scores and times are shown to have increased at the end of treatment (T1) and at follow-up (T2) in both groups. At T1 and T2, the intervention group shows significantly improved WMFT scores and times relative to those in the control group. (**A**) WMFT score, (**B**) WMFT time. WMFT: Wolf Motor Function Test. * *p* < 0.05.

The pre-treatment (T0) scores on the WMFT and time taken to perform the test were not significantly different between the intervention and control groups. When analyzed by repeated-measures ANOVA, the WMFT scores showed a significant interaction effect between time (pre-treatment vs. post-treatment) and group (intervention vs. control) (*p* = 0.012, F = 42.582) (Figure 3A).

In the intervention group, the WMFT scores increased from 23.4 ± 4.1 to 34.5 ± 5.2 after 1 month of robotic therapy (*p* = 0.004) and significantly improved to 44.2 ± 6.6 at follow-up (*p* = 0.004). In the control group, the WMFT scores significantly improved from 24.0 ± 4.5 to 30.8 ± 4.9 after rehabilitation (*p* = 0.008) and had further improved to 38.9 ± 5.7 at the follow-up (*p* = 0.010). Post hoc tests confirmed a significant difference in the WMFT scores between the intervention and control groups at T1 and T2 (*p* = 0.018 and *p* = 0.024, respectively).

Similar to the WMFT scores, a significant interaction effect between time and group was observed for WMFT time (*p* = 0.010, F = 42.582) (Figure 3B). After 1 month of treatment, the WMFT time decreased significantly in both groups, from 82 ± 13 s to 62 ± 10 s in the intervention group (*p* = 0.001) and from 81 ± 12 s to 68 ± 10 s in the control group (*p* = 0.002) at T1. At T2, the WMFT time further decreased from 62 ± 10 to 43 ± 7 s in the intervention group (*p* = 0.001) and decreased from 68 ± 10 to 51 ± 8 s in the control group (*p* = 0.004). At T1 and T2, a significant difference in WMFT time was observed between the intervention and control groups (*p* = 0.012 and *p* = 0.018, respectively).

### 3.4. Secondary Outcome Measures

**Figure 4:**
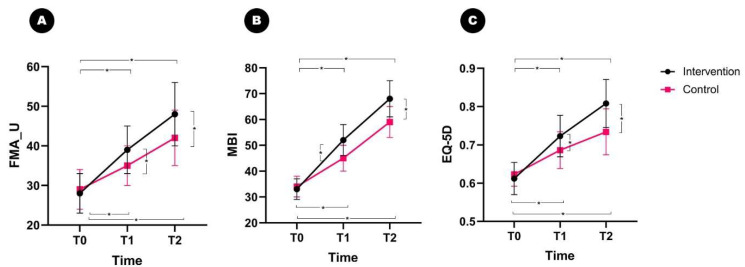
Changes in the FMA_U, MBI, and EQ-5D scores after treatment with a magnetic force hand-rehabilitation robot. The FMA_U, MBI, and EQ-5D increased significantly by the end of treatment (T1) and at follow-up (T2) in the intervention and control groups relative to the scores before the intervention start. The FMA_U, MBI, and EQ-5D scores significantly improved at T1 and T2 in the intervention group relative to those scores in the control group. (**A**) FMA_U, (**B**) MBI, (**C**) EQ-5D. FMA_U, Upper-limb score of the Fugl–Meyer Assessment; MBI, Modified Barthel Index; EQ-5D, European Quality of Life Five Dimensions. * *p* < 0.05.

No significant differences were observed between the intervention and control groups in the pre-intervention baseline scores (T0) of the FMA_U, MBI, and EQ-5D. For the FMA_U, rm-ANOVA analysis confirmed a significant interaction effect between time and group (*p* = 0.014, F = 27.423) (Figure 4A).

In the intervention group, the FMA_U increased from 28.5 ± 4.8 to 39 ± 5.6 after 1 month of treatment (*p* = 0.001) and improved to 48.5 ± 7.6 at T2 (*p* = 0.002). The control group improved from 29.2 ± 5.1 to 35.8 ± 6.0 (*p* = 0.006), and improved to 42.9 ± 6.4 at T2 (*p* = 0.012). At both T1 and T2, a significant difference in FMA_U was observed between the intervention and control groups (*p* = 0.024 and *p* = 0.032).

When analyzing the MBI, a significant interaction effect between time and group was found (*p* = 0.026, F = 32.487) (Figure 4B). In the intervention group, MBI increased from 33.3 ± 4.9 to 52 ± 6.9 after 1 month of treatment (*p* = 0.004) and improved to 68.3 ± 7.2 at T2 (*p* = 0.006). The MBI in the control group improved from 34.4 ± 5.0 to 45.3 ± 5.9 (*p* = 0.010), and improved to 59.1 ± 7.7 at T2 (*p* = 0.016). Post hoc tests confirmed a significant difference in the MBI scores between the intervention and control groups at T1 and T2 (*p* = 0.032 and *p* = 0.042).

Similarly, a significant interaction effect between time and group was observed for the EQ-5D score (*p* = 0.014, F = 36.829) (Figure 4C). In the intervention group, the EQ-5D scores increased from 0.612 ± 0.042 to 0.723 ± 0.054 after 1 month of treatment (*p* = 0.004) and improved to 0.808 ± 0.063 at T2 (*p* = 0.004). The EQ-5D scores in the control group improved from 0.623 ± 0.031 to 0.686 ± 0.048 (*p* = 0.032) and from T2 to 0.734 ± 0.060 (*p* = 0.042). At both T1 and T2, a significant difference in FMA_U score was observed between the intervention and control groups (*p* = 0.032 and *p* = 0.048).

## 4. Discussion

The use of the 3D magnetic force-driven hand-rehabilitation robot led to greater improvement in hand motor function and dexterity in the subacute patients with stroke who received 1 month of treatment than in the patients who received conventional occupational therapy. The patients treated with the robotic device continued to experience sustained recovery of paralyzed hand function 1 month after treatment completion and had better long-term results than those observed for the patients given conventional therapy. In addition, the magnetic force-based hand-rehabilitation robot not only effectively restored hand function, but also more effectively improved the independence of activities of daily living (ADLs) and health-related quality of life in the long run.

Magnetic force-driven hand rehabilitation robot has several advantages in performing rehabilitation treatment. It deployed a simpler mechanism that uses a coil and a permanent magnet to induce the movement of a paralyzed hand [16]. This makes it possible that they are easy to manufacture and that the robot is relatively small. It also has the advantage of being simple to apply this robot to rehabilitation therapy. Placing a looped magnet on the patient’s finger and inserting the hand into the robot may induce hand movements [17]. We hypothesized that this convenience would allow patients to use the device to participate collaboratively in rehabilitation therapy. Furthermore, the exercises including finger flexion and extension can help the patient make the most of the remaining muscle strength. Existing robots have a fixed axis, which limits the variety of treatment options available to patients and physical and occupational therapists. This robot only requires to synchronize the user’s desired movement to the direction of the magnetic force. In addition to simple gripping and stretching movements, it is possible to perform various hand gesture exercises such as simple adduction, twisting, and touching with both hands.

Magnetic force-driven hand rehabilitation robot can be easily linked to telerehabilitation (TR). TR means providing rehabilitation services via information and communication technologies, including video/teleconferencing, remote data-collection equipment, telemonitoring, computers, mobile phones, robotics devices, exergames, virtual reality (VR) tailored to individuals with disabilities, their families, clinicians, supervisors, and the community [21]. Several studies to improve post-stroke motor weakness of the upper extremity proved the feasibility of TR for stroke survivors [22,23,24]. They showed similar therapeutic effects compared to conventional rehabilitation, enhancing motivation and engagement and leading to sustainable recovery [22,23,24]. Our following research will combine VR and artificial intelligence technology with magnetic force-driven hand rehabilitation robots and enable stroke patients to receive hand rehabilitation treatment at home.

To date, research on the brain plasticity mechanisms involved in robotic rehabilitation to effectively improve hand and finger function in patients with stroke remains limited. Cramer et al. [25] used functional MRI to investigate the changes in brain function after robotic-assisted finger therapy in patients with stroke. This study reported a significant correlation between hand-function recovery and the total cortical sensory system, including the functional connectivity between the cortical sensory system function and the ipsilesional primary motor cortex and supplementary sensory cortex [25]. In a study that used transcranial magnetic stimulation (TMS) to investigate the mechanisms contributing to the recovery of hand function by an electromechanical robotic exoskeleton, hand-rehabilitation robotic therapy reportedly increased ipsilesional hemispheric cortical excitability [26]. Patel et al. [14] also used TMS to investigate the brain-network mechanisms involved in the effects of robotic-based upper-limb training, and similar to the above findings, reported that robotic hand rehabilitation further activated cortical reorganization. Notably, this effect was greatest immediately after the end of the 1-month treatment, and they reported that the effects of increasing neural plasticity persisted for ≤6 months [14]. The magnetic force-based hand-rehabilitation robot can apply a variety of task-oriented finger-rehabilitation programs, including finger flexion and extension, and it is speculated that it restored hand function by activating neural plasticity in the motor and sensory cortex, similar to past studies.

Patients treated with the 3D magnetism-based hand-rehabilitation robot showed improvements in ADLs and health-related quality of life, and the effects were sustained for ≤1 month after treatment completion. Hand function is closely related to the performance of ADLs, and deficits in hand function have a significant effect on health-related quality of life [7]. Studies investigating the therapeutic effect on motor function after robot-assisted finger-hand rehabilitation in patients with stroke have emphasized the need to use a combination of tools to analyze treatment effects that can adequately assess the degree of hand function in daily life, such as QuickDASH and WMFT, in addition to tools that measure upper-extremity muscle strength, such as the FMA_U and Motricity Index, which measure motor function [27,28]. ADLs are largely dependent on hand function, especially for personal activities, such as feeding, dressing, and grooming [29]. One year after a stroke, hand-function impairment reportedly was associated with reduced awareness of anxiety, health-related quality of life, and subjective well-being [30]. The 3D magnetic force-based hand-rehabilitation robot helped restore hand function, increasing the independence of patients with stroke and ultimately improving their health-related quality of life.

Various robots have been developed to restore upper limb dysfunction, including impaired hand function, after stroke, but no studies have yet been presented comparing the therapeutic effects of rehabilitation robots. Several studies that have reported the effectiveness of robotic rehabilitation using WMFT, the primary outcome used in this study, may help address these issues. Takebayashi et al. [31] provided robot-assisted self-training for stroke patients using the ReoGo upper extremity system. WMFT scores and time of the patients were significantly improved when the robotic rehabilitation was administered for six weeks of rehabilitation treatment [31]. The effect of robotic rehabilitation was more pronounced in patients with severe motor deficits with a Fugl-Meyer Assessment (FMA) score of less than 30 [31]. This system is complicated to compare directly with the robot in this study because it focuses on proximal shoulder girdle training rather than hand and finger. Still, it has contributed to robotic rehabilitation therapy and improvement of upper limb function, similar to this study.

Another study presented a rehabilitation assessment robot using artificial intelligence with a close correlation with WMFT [32]. When the upper limb function was evaluated using the robot developed by applying the Back Propagation Neural Network model, it was reported that the accuracy reached 87.1% compared to the WMFT performed by the human assessor [32]. Wolf et al. [33] investigated whether restoring upper limb motor function after a stroke is possible by providing a home rehabilitation program remotely using the Hand Mentor system. Hand Mentor is a robot that can perform hand grip exercises using a pneumatic actuator [33]. Both patients who received remote robotic upper limb rehabilitation and those who self-exercised with a remotely delivered program without a robot reported improved upper limb function when assessed using WMFT [33]. It is speculated that the results of each study are different because the patients in each study are heterogeneous, and each robot has another mechanism for providing rehabilitation. In addition, not only the performance of the robot but also the rehabilitation program using the robot can affect the degree of recovery. More research is needed on robotic rehabilitation protocols that can more effectively improve upper limb function, including hands and technologies that use robots to assess hand function in the future.

This study had some limitations. Although the developed robot demonstrated therapeutic effects on hand-function recovery immediately after treatment, the recovery mechanism was not investigated. Research using various tools, such as functional MRI, on the mechanisms underlying the ability of robot-assisted rehabilitation to induce brain-network reorganization and enhance brain neuroplasticity remain limited. In addition, the robotic hand-rehabilitation protocol in our study was developed by physicians and occupational therapists with extensive clinical experience in stroke rehabilitation. However, the method of rehabilitation with robotics itself may also affect the results. These limitations should be addressed in future studies.

## 5. Conclusions

Rehabilitation using the 3D hand-rehabilitation robot contributed to the recovery of hand function in subacute patients with stroke and showed therapeutic effects. The 3D hand-rehabilitation robot has the advantage of being able to apply various rehabilitation training programs because it uses a magnetic field to help the patients execute desired movement regardless of the hand position and then implements the finger movements desired by the therapist. In the long term, hand-function restoration therapy increased the independence in performing ADLs and improved the quality of life of patients with stroke. The 3D magnetic force-based hand-rehabilitation robot is expected to be useful in clinical rehabilitation programs aimed at enhancement of hand function in patients with stroke.

## Figures and Tables

**Table 1:** Demographic characteristics of the participants.

		Intervention Group (n = 18)	Control Group (n = 18)
Age	(years, mean ± SD)	60.5 ± 7.8	61.3 ± 8.4
Sex	Male	8	9
	Female	10	9
Stroke type	Infarct	13	13
	Hemorrhage	5	5
Dominant hand	Right	16	16
	Left	2	2
Affected side	Right	6	6
	Left	12	12
Period after stroke onset	(days, mean ± SD)	29.1 ± 6.2	29.6 ± 7.2
NIHSS score	(onset)	8.2 ± 3.4	8.8 ± 4.1
Spasticity (MAS)	0	8	9
	1	9	9
	2	1	0
MoCA score	(mean ± SD)	17.2 ± 3.3	17.0 ± 3.8
Comorbidity	Hypertension	16	16
	Diabetes	11	10

NIHSS, National Institutes of Health Stroke Scale; MAS, Modified Ashworth Scale; MoCA, Montreal Cognitive Assessment; SD, Standard deviation.

## Data Availability

The datasets analyzed during the current study are available from the corresponding author on reasonable request. The data are not publicly available due to privacy or ethical restrictions.

## References

[1] Schieber M.H., Santello M. (2004). Hand function: Peripheral and central constraints on performance. J. Appl. Physiol..

[2] Exner C.E., Henderson A., Pehoski C. (2006). Chapter 12—Intervention for Children with Hand Skill Problems. Hand Function in the Child.

[3] Langhorne P., Coupar F., Pollock A. (2009). Motor recovery after stroke: A systematic review. Lancet Neurol..

[4] Pollock A., Farmer S.E., Brady M.C., Langhorne P., Mead G.E., Mehrholz J., van Wijck F. (2014). Interventions for improving upper limb function after stroke. Cochrane Database Syst. Rev..

[5] Hayward K.S., Kramer S.F., Dalton E.J., Hughes G.R., Brodtmann A., Churilov L., Cloud G., Corbett D., Jolliffe L., Kaffenberger T. (2021). Timing and Dose of Upper Limb Motor Intervention After Stroke: A Systematic Review. Stroke.

[6] Cha T.H., Hwang H.S. (2022). Rehabilitation Interventions Combined with Noninvasive Brain Stimulation on Upper Limb Motor Function in Stroke Patients. Brain Sci..

[7] Lee G.S., Kim S.H., Ji D.M., Kong D.H., Jung Y.J., Joo M.C., Yun N.R., Soh S.-H., Park J.W., Kim M.-S. (2019). Feasibility and Therapeutic Effects of a Novel Magnet-Based Device for Hand Rehabilitation: A Pilot Study. Brain Neurorehabilit..

[8] Yue Z., Zhang X., Wang J. (2017). Hand Rehabilitation Robotics on Poststroke Motor Recovery. Behav. Neurol..

[9] Lee S.H., Park G., Cho D.Y., Kim H.Y., Lee J.Y., Kim S., Park S.B., Shin J.H. (2020). Comparisons between end-effector and exoskeleton rehabilitation robots regarding upper extremity function among chronic stroke patients with moderate-to-severe upper limb impairment. Sci. Rep..

[10] Morone G., Palomba A., Martino Cinnera A., Agostini M., Aprile I., Arienti C., Paci M., Casanova E., Marino D., LA Rosa G. (2021). Systematic review of guidelines to identify recommendations for upper limb robotic rehabilitation after stroke. Eur. J. Phys. Rehabil. Med..

[11] Lee B.O., Saragih I.D., Batubara S.O. (2023). Robotic arm use for upper limb rehabilitation after stroke: A systematic review and meta-analysis. Kaohsiung J. Med. Sci..

[12] Ko M.J., Chuang Y.C., Ou-Yang L.J., Cheng Y.Y., Tsai Y.L., Lee Y.C. (2023). The Application of Soft Robotic Gloves in Stroke Patients: A Systematic Review and Meta-Analysis of Randomized Controlled Trials. Brain Sci..

[13] Kang B.B., Choi H., Lee H., Cho K.J. (2019). Exo-Glove Poly II: A Polymer-Based Soft Wearable Robot for the Hand with a Tendon-Driven Actuation System. Soft Robot..

[14] Patel J., Fluet G., Qiu Q., Yarossi M., Merians A., Tunik E., Adamovich S. (2019). Intensive virtual reality and robotic based upper limb training compared to usual care, and associated cortical reorganization, in the acute and early sub-acute periods post-stroke: A feasibility study. J. Neuroeng. Rehabil..

[15] Gu Y., Xu Y., Shen Y., Huang H., Liu T., Jin L., Ren H., Wang J. (2022). A Review of Hand Function Rehabilitation Systems Based on Hand Motion Recognition Devices and Artificial Intelligence. Brain Sci..

[16] Ji D.-M., Kim M.-S., Kim S.-H. (2021). Multi-Link Magnet Device with Electromagnetic Manipulation System for Assisting Finger Movements with Wireless Operation. Appl. Sci..

[17] Kim S.H., Ji D.M., Kim C.Y., Choi S.B., Joo M.C., Kim M.S. (2022). Therapeutic Effects of a Newly Developed 3D Magnetic Finger Rehabilitation Device in Subacute Stroke Patients: A Pilot Study. Brain Sci..

[18] Baek I.-C., Kim M., Kim S. (2017). A Novel Nonmechanical Finger Rehabilitation System Based on Magnetic Force Control. J. Magn..

[19] Karamians R., Proffitt R., Kline D., Gauthier L.V. (2020). Effectiveness of Virtual Reality- and Gaming-Based Interventions for Upper Extremity Rehabilitation Poststroke: A Meta-analysis. Arch. Phys. Med. Rehabil..

[20] Hodics T.M., Nakatsuka K., Upreti B., Alex A., Smith P.S., Pezzullo J.C. (2012). Wolf Motor Function Test for characterizing moderate to severe hemiparesis in stroke patients. Arch. Phys. Med. Rehabil..

[21] Nikolaev V.A., Nikolaev A.A. (2022). Recent trends in telerehabilitation of stroke patients: A narrative review. NeuroRehabilitation.

[22] Chen Y., Chen Y., Zheng K., Dodakian L., See J., Zhou R., Chiu N., Augsburger R., McKenzie A., Cramer S.C. (2020). A qualitative study on user acceptance of a home-based stroke telerehabilitation system. Top. Stroke Rehabil..

[23] Cramer S.C., Dodakian L., Le V., See J., Augsburger R., McKenzie A., Zhou R.J., Chiu N.L., Heckhausen J., Cassidy J.M. (2019). Efficacy of Home-Based Telerehabilitation vs In-Clinic Therapy for Adults After Stroke: A Randomized Clinical Trial. JAMA Neurol..

[24] Szturm T., Imran Z., Pooyania S., Kanitkar A., Mahana B. (2021). Evaluation of a Game Based Tele Rehabilitation Platform for In-Home Therapy of Hand-Arm Function Post Stroke: Feasibility Study. PM&R.

[25] Ingemanson M.L., Rowe J.R., Chan V., Wolbrecht E.T., Reinkensmeyer D.J., Cramer S.C. (2019). Somatosensory system integrity explains differences in treatment response after stroke. Neurology.

[26] Singh N., Saini M., Kumar N., Srivastava M.V.P., Mehndiratta A. (2021). Evidence of neuroplasticity with robotic hand exoskeleton for post-stroke rehabilitation: A randomized controlled trial. J. Neuroeng. Rehabil..

[27] Moggio L., de Sire A., Marotta N., Demeco A., Ammendolia A. (2022). Exoskeleton versus end-effector robot-assisted therapy for finger-hand motor recovery in stroke survivors: Systematic review and meta-analysis. Top. Stroke Rehabil..

[28] Yang X., Shi X., Xue X., Deng Z. (2023). Efficacy of Robot-Assisted Training on Rehabilitation of Upper Limb Function in Patients with Stroke: A Systematic Review and Meta-Analysis. Arch. Phys. Med. Rehabil..

[29] Pournajaf S., Morone G., Straudi S., Goffredo M., Leo M.R., Calabrò R.S., Felzani G., Paolucci S., Filoni S., Santamato A. (2023). Neurophysiological and Clinical Effects of Upper Limb Robot-Assisted Rehabilitation on Motor Recovery in Patients with Subacute Stroke: A Multicenter Randomized Controlled Trial Study Protocol. Brain Sci..

[30] Rosenfeldt A.B., Linder S.M., Davidson S., Clark C., Zimmerman N.M., Lee J.J., Alberts J.L. (2019). Combined Aerobic Exercise and Task Practice Improve Health-Related Quality of Life Poststroke: A Preliminary Analysis. Arch. Phys. Med. Rehabil..

[31] Takebayashi T., Takahashi K., Okita Y., Kubo H., Hachisuka K., Domen K. (2022). Impact of the robotic-assistance level on upper extremity function in stroke patients receiving adjunct robotic rehabilitation: Sub-analysis of a randomized clinical trial. J. NeuroEng. Rehabil..

[32] Zhang M., Chen J., Ling Z., Zhang B., Yan Y., Xiong D., Guo L. (2022). Quantitative Evaluation System of Upper Limb Motor Function of Stroke Patients Based on Desktop Rehabilitation Robot. Sensors.

[33] Wolf S.L., Sahu K., Bay R.C., Buchanan S., Reiss A., Linder S., Rosenfeldt A., Alberts J. (2015). The HAAPI (Home Arm Assistance Progression Initiative) Trial: A Novel Robotics Delivery Approach in Stroke Rehabilitation. Neurorehabilit. Neural Repair.

